# Electrochemical Detection of *E. coli* O157:H7 in Water after Electrocatalytic and Ultraviolet Treatments Using a Polyguanine-Labeled Secondary Bead Sensor

**DOI:** 10.3390/s18051497

**Published:** 2018-05-10

**Authors:** Michael G. Beeman, Ugochukwu C. Nze, Himanshu J. Sant, Hammad Malik, Swomitra Mohanty, Bruce K Gale, Krista Carlson

**Affiliations:** 1 Department of Mechanical Engineering, University of Utah, Salt Lake City, UT 84112, USA; u.c.nze@utah.edu (U.C.N.); bruce.gale@utah.edu (B.K.G.); 2 Espira Inc., 825 N 300 W Suite N-223, Salt Lake City, UT 84103, USA; 3 Department of Metallurgical Engineering, University of Utah, Salt Lake City, UT 84112, USA; m.malik.hammad@gmail.com (H.M.); swomitra@chemeng.utah.edu (S.M.); krista.carlson@utah.edu (K.C.); 4 Department of Chemical Engineering, University of Utah, Salt Lake City, UT 84112, USA

**Keywords:** *Escherichia coli* O157:H7 detection, defect laden titania (TiO_2_)-based reactor, biosensors, pathogen detection, electrochemical detection, square wave voltammetry, immunomagnetic separation

## Abstract

The availability of clean drinking water is a significant problem worldwide. Many technologies exist for purifying drinking water, however, many of these methods require chemicals or use simple methods, such as boiling and filtering, which may or may not be effective in removing waterborne pathogens. Present methods for detecting pathogens in point-of-use (POU) sterilized water are typically time prohibitive or have limited ability differentiating between active and inactive cells. This work describes a rapid electrochemical sensor to differentially detect the presence of active *Escherichia coli (E. coli)* O157:H7 in samples that have been partially or completely sterilized using a new POU electrocatalytic water purification technology based on superradicals generated by defect laden titania (TiO_2_) nanotubes. The sensor was also used to detect pathogens sterilized by UV-C radiation for a comparison of different modes of cell death. The sensor utilizes immunomagnetic bead separation to isolate active bacteria by forming a sandwich assay comprised of antibody functionalized secondary magnetic beads, *E. coli* O157:H7, and polyguanine (polyG) oligonucleotide functionalized secondary polystyrene beads as an electrochemical tag. The assay is formed by the attachment of antibodies to active receptors on the membrane of *E. coli*, allowing the sensor to differentially detect viable cells. Ultravioloet (UV)-C radiation and an electrocatalytic reactor (ER) with integrated defect-laden titania nanotubes were used to examine the sensors’ performance in detecting sterilized cells under different modes of cell death. Plate counts and flow cytometry were used to quantify disinfection efficacy and cell damage. It was found that the ER treatments shredded the bacteria into multiple fragments, while UV-C treatments inactivated the bacteria but left the cell membrane mostly intact.

## 1. Introduction

Enterohemorrhagic *Escherichia coli* (*E. coli*) serotype O157:H7 is a highly infectious food and waterborne bacteria that effects populations worldwide [1]. Antimicrobial use against *E. coli* O157:H7 causes the cell to release Shiga toxins that cause bloody diarrhea, and in some severe cases, hemolytic uremic syndrome [1]. The infectious dose limit of *E. coli* O157:H7 has been reported to be as low as 10–100 organisms, which is less than the detection limits of many current detection methods [2]. Many developing countries without access to municipal water rely on point-of-use (POU) sterilization methods to combat this pathogen and many others [3]. However, there is still the need for a POU pathogen detection system to ensure the efficacy of the disinfection method of choice.

Plate counting is the gold standard method for detecting *E. coli* O157:H7 [4,5]. This method is not ideal for POU because it requires long incubation times (2–3 days) and is labor intensive. Another common approach identifies bacteria nucleic acids by attaching a synthetic oligonucleotide probe or primer to the complimentary target sequence for detection [6]. Polymerase chain reaction (PCR) is used to amplify Shiga toxin producing *Escherichia* coli (STEC) genes by replicating the gene sequence. PCR is highly specific, allowing for the detection of a single DNA strain, and can be employed for real time presence/absence and quantification tests. Although highly effective at detecting the presence of STEC, PCR methods only detect the presence of bacteria and, do not directly differentiate between viable cells capable of producing Shiga toxins and inactivated non-pathogenic bacteria. A drawback to all of these methods is that the samples must be sent to sterile laboratories containing expensive and bulky equipment, managed by skilled labor [7]. These methods are cost and time restrictive for most POU users who require expedient results in a few hours, instead of days [8,9]. People living in developing nations are less likely to have access to these resources [10].

Antibody based methods are also utilized in detection through the use of antibody–antigen binding to identify and separate antigens from a sample. Enzyme-linked immunosorbent assay (ELISA) is commonly used for the detection of foodborne pathogens [6]. ELISA is a relatively fast and sensitive method for the detection of *E. coli*. For example, Shen et al. demonstrated the use of ELISA to detect 68 cfu/mL of *E. coli* O157:H7 in 3 h using beacon gold nanoparticles [11]. Detection methods described in this study are similar to ELISA, but use a sandwich assay between a primary antibody-coated magnetic bead, an antigen, and a secondary antibody-coated polystyrene bead.

Jayamohan et al. demonstrated the detection system reported in this paper, which is comprised of an immunomagnetic bead separation system and an electrochemical sensor used to detect the presence of *E. coli* O157:H7 [12,13]. Using this method, Jayamohan et al. was able to detect 3 cfu/mL in two hours using immunomagnetic bead separation paired with an electrochemical sensor [12]. This detection method was over 22 times more sensitive, and was performed in much less time compared to ELISA [12]. This technology has potential to be paired with a POU sterilization system to provide highly specific information about potential pathogens in drinking water in as short as 2 h.

In this paper, our group has combined this sensor technology with a POU waterborne pathogen disinfection system for the first time. The novel focus of this paper is to demonstrate that the electrochemical sensor could be paired with ER sterilization. The disinfection system consists of a defect laden titania nanotube-based reactor that physically destroys waterborne pathogens via the electrocatalytic generation of oxidizing radicals [14,15,16]. Cell death occurs in this electrocatalytic reactor (ER) via interaction with oxidizing radical species formed on the surface of the titania anode. Carlson et al. demonstrated that by introducing a large number of inter- and intraband defects into the TiO_2_ during annealing, oxidizing species could be generated at the TiO_2_ surface upon the application of a 3–6 V anodic bias [14]. It was found that when used as a batch reactor, cell death in the ER was a result of interactions with the holes on the TiO_2_ surface (h_VB_^+^), hydroxyl radicals (OH^•^), and hydrogen peroxide (H_2_O_2_) [14,15]. These radicals attack cells, and death occurs as a result of both physical damage to the outer cell membrane and/or structural changes within the plasma membrane [15,17]. The advantage of this methodology is that it is as effective as ozone treatment in inactivating waterborne pathogens without the need for equipment associated with typical ozone systems.

Although the pathogens that pass through the ER are killed and were no longer physically intact, the sensing technology presented can detect the presence of inactivated pathogens if receptors on the bacteria are still receptive to antibodies. In order to develop a successful POU sensor for the detection of viable pathogens in drinking water, it is imperative that the mechanism of cell death, and how that relates to the detection of the pathogen on the sensor, is understood. Even more important is the effect of the treatment on the cell membrane, as this is where the receptors of the antibodies attach to the pathogen. If the antibodies attach to an inactivated cell, forming a sandwich assay, the attached tag would indicate a positive result for the pathogen. In applications such as POU sterilization, it is not desirable for a detection system to show positive indication of the inactivated pathogens. To the best of the knowledge of these authors, there is currently no other research that considers the bactericidal effects of an electrocatalytic reactor and ultraviolet treatment on the efficacy of an electrochemical sensor of this type (i.e., the ability to differentiate between live and dead cells).

**Figure 1:**
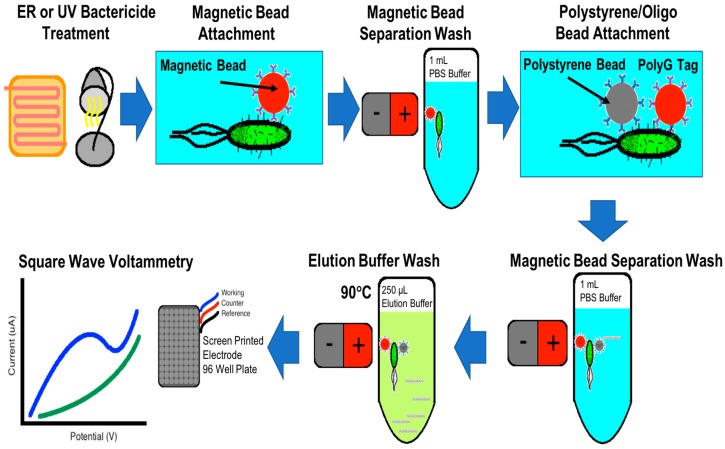
Immunomagnetic separation flowchart detailing the capture of the magnetic bead/*E. coli*/polystyrene bead oligo sandwich assay for square wave voltammetry signal detection of *E. coli*.

This work describes the use of immunomagnetic bead separation (IMS) and electrochemical sensor technology for the ultrasensitive detection of the presence of viable *E. coli* O157:H7. IMS uses a magnet to fix magnetic beads to the side of a container so that contaminants or excess reagents can be removed from the container without removing the magnetic beads. Through a series of suspension and magnetic bead separation cycles, the bacteria form a sandwich assay between antibody-conjugated magnetic beads and an antibody-conjugated polystyrene bead that is also functionalized with a synthetic polyG oligonucleotide tag. The streptavidin coated polystyrene bead is conjugated with a biotinylated polyG bio-barcode tag, and goat antibody reactive to *E. coli*, O and K antigenic serotypes. PolyG oligonucleotides were used as a bio-barcode that, when detected using electrochemical detection, give a peak current density signal at ~1 V. Figure 1 graphically represents the immunomagnetic separation and electrochemical sensor [12,13]. The use of oligonucleotide functionalized secondary beads provides amplification of the electrochemical signal in allowing multiple polystyrene beads to bind with the bacteria. After attachment, the polyG tag is separated from the polystyrene bead using an elution buffer, and quantified by measuring the oxidation current density of the sample. This paper will discuss how IMS washing steps allow the sensor to selectively separate sterilized bacteria from whole viable cells for detection on the electrochemical sensor.

## 2. Materials and Methods

### 2.1. Culturing E. coli O157:H7 Cells and Plate Counting

Culturing of *E. coli* O157:H7 bacteria obtained from American Type Culture Collection (Cat. No. 700728, ATCC, Manassas, VA, USA) was performed using the protocols provided by ATCC. The freeze-dried bacteria were reconstituted in 6 mL of Difco™ Nutrient broth (Cat. No. 234000, Becton Dickinson, Sparks, MD, USA). A 200 µL sample was plated on a Difco™ nutrient agar plate (Cat. No. 23400, Becton Dickson, Sparks, MD, USA) and incubated for 36 h at 37 °C to verify viability. After incubation, the broth was centrifuged at 1000 *g* for 10 min to form the bacteria into a pellet. Supernatant was removed, and the pellet was washed with 3 mL of broth along with 3 mL of sterilized 20% glycerol (v/v). The sample was then placed in Nalgene Cryogenic vials (Thermo Scientific, Waltham, MA, USA) and stored in a cryogenic freezer at −135 °C for later use.

Samples were prepared on DifcoTM Nutrient Agar (Cat. No. 213000, Becton Dickinson, Franklin Lakes, NJ, USA) plates and incubated at 37 °C for 24 h. Bacteria were scraped from the sample and suspended in a 1x phosphate buffer solution (PBS). A spectrophotometer (Biochrom WPA BioWave DNA Lifescience Spectrophotometer Cat. No. 80-3004-70) was used to measure the optical density of the sample at a wavelength of 600 nm (OD600). A manual hemocytometer was used to calibrate the spectrophotometer readings to colony forming units (cfu) per volume. It was determined that an OD600 of 0.1 correlates to a bacteria concentration of 50 × 10^6^ cfu/mL. The bacteria samples were diluted to an OD600 of 0.1 and then diluted in 20 ppm NaCl solution to a concentration on the order of 10 × 10^4^ cfu/mL. Concentrations were verified using the plate count method. A concentration of 20 ppm was chosen based on our previous work, that showed that 20 ppm NaCl provided enough electrolyte for the bacteria to survive, but not so much that chlorine radical production began to have a significant impact on bactericide.

The plate count method grows colonies on agar plates that relate to the number of viable cells in a solution [18]. An aliquot of 50 μL of each sample was spread onto agar plates and incubated at 37 °C for 24 h. Bacteria cells on the plate divide to form a new cell during the cells logarithmic growth phase, forming a visible colony on the plate. Each colony was counted to determine the number of colony forming cells in the sample as,

(1) $$C\;[CFU/mL]=\frac{N}{V}\times 10^{n}$$

where, *N* is the number of colonies counted on the plate, *V* is the volume of the sample added to the plate and n is the number of serial dilutions (10-fold).

### 2.2. E. coli Inactivation

*E. coli* solutions were inactivated using either the TiO_2_-based ER or ultraviolet (UV-C) sterilization. For samples treated with the ER, 10 mL of the 10 × 10^4^ cfu/mL *E. coli* solution was placed in the batch reactor for 15 s, 5 min, or 15 min. Once injected into the reactor, a 6 V anodic bias was applied to a titanium wire coil with defect laden TiO_2_ nanotubes covering the surface. A detailed description of the formation of these nanotubes via anodization is described in our previous work [14,15]. The stainless steel backplate on the reactor acted as the cathode. To clean between tests, 100 mL of distilled water was passed through the ER. Sterilization of the *E. coli* could be caused by water splitting if the potential of the system favored electrolysis [14,15]. Minimal water splitting was observed for exposure times exceeding 15 min, due to the overpotential for water oxidation of the system. The current through the system varied between 4–6 mA during operation. It should be noted that there were no noticeable bubbles formed in the reactor, indicating little, if any, water splitting.

Samples inactivated by a mercury ultraviolet (UV) lamp (Cat. no UVGL-25, UVP, Cambridge, UK) were aliquoted into 10 mL samples and placed in open 100 mL sterile cups. The samples were placed 6 inches from the lamp and exposed at a light intensity of 4 W with a peak intensity at 254 nm. Samples were exposed for 15 s, 5 min, and 15 min. A sample of 20 ppm NaCl without *E. coli* O157:H7 was used as a negative control. A sample not exposed to UV radiation or radicalized in the ER was used as the positive control. The plate count method was used to quantify the number of bacteria in each sample that were either treated, untreated, or samples containing pure buffer solution.

### 2.3. Immunomagnetic Bead Separation of E. coli

Anti-*E. coli* O157:H7 (Dynabeads, Cat. No. 71003) obtained from ThermoFisher (Carslbad, CA, USA) were added to each sample and suspended for 40 min. All samples were processed in 1 mL aliquots. Two IMS washes using the 1x PBS solution were used to rinse and remove unbound contaminants from the sample. Centrifuge tubes containing the sample were placed on a custom-made magnetic bead rack for 3 min. The samples were rotated on the rack after 1.5 min to improve capture. 1 mL of 1x PBS buffer solution was used to wash. Between each wash, samples were vortex-mixed to remove any whole or partial bacteria cells that were nonspecifically bound to the walls or magnetic beads. Secondary fluorescent polystyrene beads conjugated with anti-*E. coli* antibodies were then suspended in the sample. The polystyrene beads were conjugated with a synthetic polyG oligo electrochemical tag. An IMS wash was repeated to remove any unattached secondary beads.

### 2.4. Conjugation of Polystyrene Beads with Flourescent Dyes

Streptavidin coated 1 µm diameter 10 mg/mL microspheres (Cat. No. CP01F, Bangs Labs, Fishers, IN, USA) were used as a secondary bead in the magnetic bead/*E. coli*/polystyrene bead assay. An aliquot of 20 µL of polystyrene beads suspended in storage buffer were centrifuged at 5 rcf for 5 min to form a pellet. The supernatant was removed, and the pellet was resuspended in 1 mL of 1x PBS solution. This wash was performed twice to remove the storage buffer. The washed pellet was suspended in 2 µL of 4–5 mg/mL anti-*E. coli* antibody (Cat. No. B65109B, Meridian Life Science, Inc., Memphis, TN, USA) and 998 µL of 1x PBS solution on a rotator for 2 h. A centrifuge wash was repeated, and the pellet was resuspended in 20 µL of 1x PBS solution and 1.53 µL of 500 µM biotinylated polyG (GGGGGGGGGGGGGGGGGGGG/30-Biotin). The biotinylated antibody was conjugated to the polystyrene bead forming a streptavidin/biotin bond. The oligonucleotides were obtained from the DNA/Peptide synthesis core facility (University of Utah, Salt Lake City, UT, USA).

### 2.5. Electrochemistry Measurements

After the second IMS wash, 250 μL of elution buffer comprised of 5 vol % DNase/RNase free UltraPure™ water (Invitrogen by Life Science, Waltham, MA, USA), 45 vol % formamide (Thermo Scientific, Waltham, MA, USA) and 50 vol % 80 mM NaOAc (sodium acetate) was used to remove the polyG oligos from the bead assay. Sodium acetate was used as an electron mediator during the oxidation of polyG. Samples containing the elution buffer and beads were heated to 90 °C for 10 min, and then transferred to a three-electrode 96 well plate (Cat. No. DRP-96x550, DropSens, Asturias, Spain) placed in a Faraday cage. The working, counter, and reference electrodes were comprised of carbon, carbon and silver, respectively. Square wave voltammetry (SWV) was used to detect the redox current density of the polyG oligo tag. An EmStat3+ potentiometer, along with PalmSens (PsTrace v5.3) software, was used to measure the current density of the redox reaction using square wave voltammetry (SWV). SWV measurements were taken between 0.4–1.2 V at a scan rate of 500 mVs^−1^.

### 2.6. Flow Cytometry

**Figure 2:**
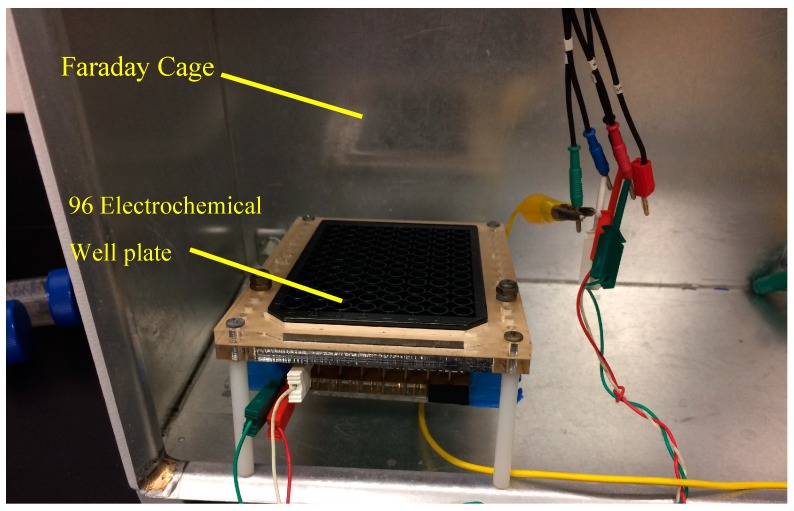
Experimental setup displaying the Faraday cage and custom electrochemical well plate reader.

In addition to electrochemical measurements, samples were analyzed using multivariable statistics utilizing a BD FACSCANTO II flow cytometer. *E. coli* samples containing 5 × 10^6^ cfu/mL were stained to a concentration of 1:20 Hoechst 33342 dye (ThermoFisher Carslbad, CA, USA), and incubated at 37 °C for 10 min so that bacteria could be distinguished from particulates. *E. coli* concentrations were increased to 5 × 10^6^ for flow cytometer experiments so that the signal of whole *E. coli* was comparable to particulates found in the PBS buffer solution. The samples were prepared and treated in the ER using the same protocol previously outlined. Each test was performed in triplicate to verify repeatability. Gates in the flow cytometer were set to compare the side scatter of the population versus an Alexa Fluor 405-A signature. This enables dyed *E. coli* to be distinguished from similar but un-dyed debris particles in a population. Figure 2 displays the experimental setup.

### 2.7. Scanning Electron Microscopy

Scanning electron microscopy (SEM) was performed using a FEI Quanta 600 FEG. For sample preparation, the solution containing the bacteria to be examined was filtered through 0.25 m filter paper. The filter paper was dried overnight in a vacuum drying oven at 27 °C, and subsequently placed on an SEM stub for imaging. The samples were left uncoated as low-vacuum mode was used for imaging.

## 3. Results and Discussion

### 3.1. Pathogen Disinfection

Exposure of *E. coli* to ER and UV treatments both resulted in cell death, as shown from the averaged plate counts and averaged peak signals in Table 1 and Table 2, although death occurred from different inactivation mechanisms. The peak current density of each signal was compared to the control samples, original and blank, to quantify sterilization. A reduction in polyG signal correlates to the length of exposure time of UV-C radiation or oxidation from the ER with peaks occurring at approximately 0.98–1.10 V. It was determined from a comparison of plate counts with sensor peak signals in Table 1 and Table 2 that the sensor can differentially detect live bacteria. ER treatments resulted in the complete destruction of the cell membrane after 15 s, while only partial inactivation occurred for samples receiving UV-C radiation dosage. This study averaged three samples and reports standard error for each set of samples, presented in Table 1 and Table 2. Statistical comparison between averaged peak signals at each control sample were done by performing a Student *t* test to evaluate the null hypothesis between original/no treatment and blank samples. Probability values of 0.12 and 0.10 were found for ER and UV, respectively. For this feasibility study, this confidence criterion was deemed acceptable. It is apparent from the variability of peak current density signals that multiple samples are required to obtain average peak signal values not overly influenced by outliers.

**Figure 3:**
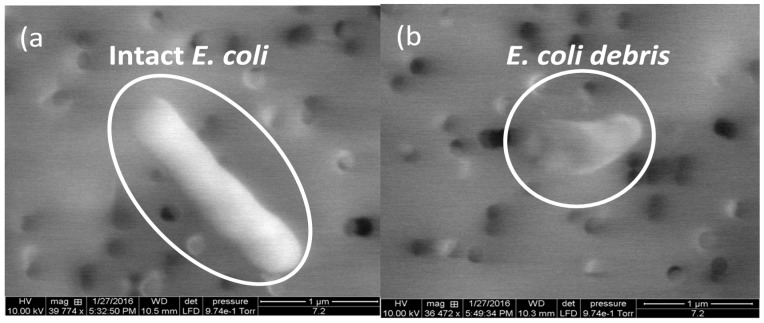
An example of SEM images of *E. coli* cells (**a**) before and (**b**) after electrocatalytic reactor treatments.

In the ER, the mechanism of disinfection is through oxidation, which results in physical damage to the cell membrane. Physical destruction of the cells verified through SEM images of the original *E. coli* cells (Figure 3a), and cells after a 15 s treatment in the ER (Figure 3b). Only small fragments could be observed after a 15 min treatment with the ER.

Physical damage and *E. coli* death in the ER occur due to cell reactions with radical species generated at the surface of TiO_2_. When an anodic bias is applied to the TiO_2_-based electrode, valence band (VB) electrons are excited to the conduction band, creating “holes” within the VB (h_VB+_). If in an aqueous solution, these holes react with adsorbed water and/or hydroxyl ions for the direct production of hydroxyl radical (OH^•^) at the surface of the TiO_2_, as shown in Equation (1) [19]:

Ti^IV^O_2_ − H_2_ O_ads_ + (h_VB_^+^) → Ti^IV^O_2_ + H^+^ + OH (2)

Other reactive oxygen species (e.g., superoxide (O_2_^•−^), hydroperoxyl (^•^OOH)) may also form and contribute to the oxidative pathways. These radicals can form through interactions with electrons or with interband states (e.g., Ti^3+^) present in the TiO_2_ [19,20]. Equation (2) shows an example of this process for oxygen reacting with Ti^3+^ to form O_2_^•−^ [19]

Ti^III^O_2_ + O_2_ → Ti^IV^O_2_ + O_2_^•−^ (3)

These radical species play a key role in the disinfection of pathogens using TiO_2_. However, surface OH^•^ is the main contributor to disinfection, as it has the highest oxidation potential compared to the other species.

UV sterilization is a result of high energy photons breaking down organic material causing damage to the nucleic acid, that does not directly kill the cell, but prevents it from replicating [21,22]. UV treatment does not lyse the internal cell membrane, but can cause disruption to nucleic acids within the membrane. UV radiation breaks apart genetic base pairs, resulting in the misincorporation of DNA, or DNA lesions, during reproduction [22]. The literature has shown distortion of bacteria shape, and also likely affects membrane receptors. If the DNA is not correctly repaired, programmed mechanisms in the bacteria lead directly to cell apoptosis. Additionally, if sufficient damage is done to the external and internal membrane, DNA leakage will occur. If the DNA is not correctly repaired, programmed mechanisms direct the cell to apoptosis. Liu et al. reported nanoscale images of *E. coli* exposed to UV light, displaying whole cells that were twisted and had rough surfaces when compared to untreated cells [21]. Damage to the outer membrane wall would also affect the ability of the cell’s receptors to bind to anti-*E. coli* antibodies.

**Figure 4:**
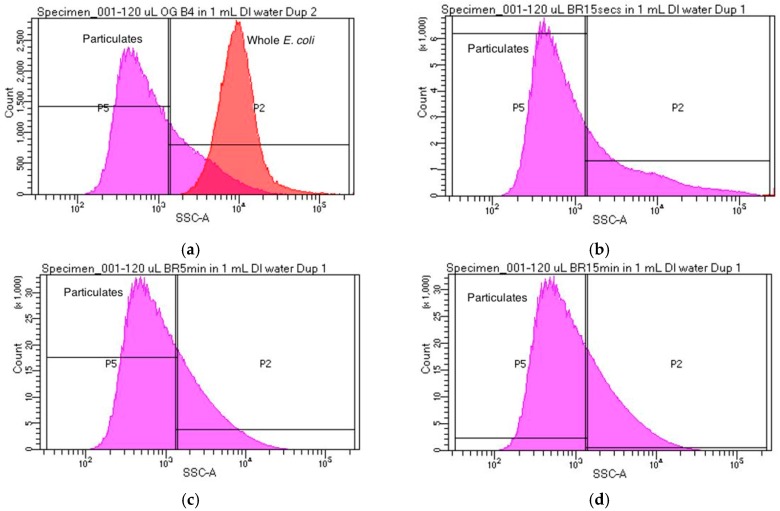
Population side scatter distribution for each sample tested. In each plot, the curve labeled “Whole *E. coli*” corresponds to the side scatter distribution of whole *E. coli* cells, while the curve labeled “Particulates” corresponds to debris and other particulates that are not whole *E. coli* cells. (**a**) Side scatter distribution for the original sample which did not undergo treatment with the ER; (**b**) Side scatter distribution for a sample which underwent 15 s treatment with the ER; (**c**) Side scatter distribution for a sample which underwent 5 min treatment with the ER; (**d**) Side scatter distribution for a sample which underwent a 15 min treatment with the ER.

Flow cytometry experimental results in this work also confirm the morphology of *E. coli* cells after ER and UV treatments. Population side scatter histogram distributions were used to categorize cells as either whole *E. coli*, shredded *E. coli*, or debris. Each sample was analyzed using flow cytometry and population side scatter (SSC) distributions were recorded. Figure 4a illustrates the SSC distribution for the original sample containing untreated *E. coli*. Figure 4b displays SSC distribution of the population after a 15 s ER treatment. Note the absence of cells with the same SSC correlating to whole *E. coli* cells. The total amount of debris particulates in Figure 4b (347,174 counts) more than double the total debris counts from Figure 4a (129,324 counts) at the most prominent SSC measurement. This is indicative of a drastic reduction in whole *E. coli* cells, and an increase in particulates from these shredded parent cells. Similar trends can be found in samples that underwent 5 and 15 min treatments of the ER (Figure 4b,c). It was observed that in these samples, the particulate count rose to over 1,920,473 with almost no particles displaying the same side scatter as whole *E. coli* cells. This displays the complete destruction of all whole *E. coli* cells after a 5 min treatment with the ER. Flow cytometry results correlate with plate count and electrochemical detection. Sterilization was observed for cells receiving ER treatment using plate count and electrochemical detection as well.

**Figure 5:**
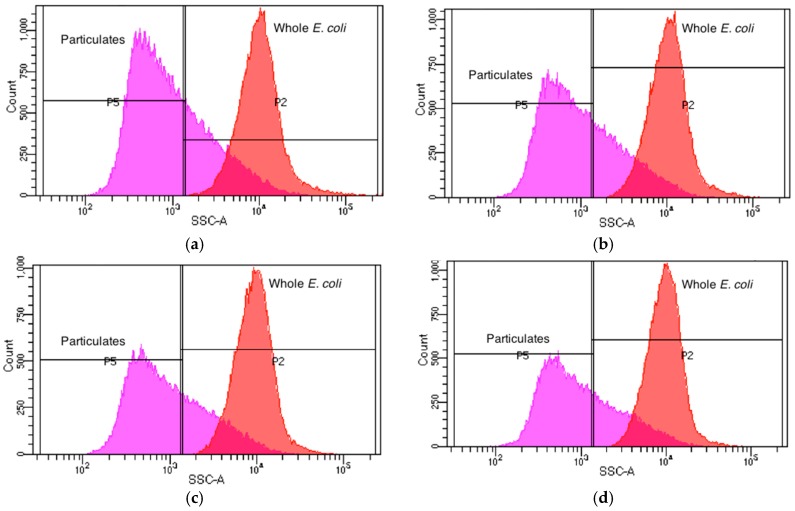
Population side scatter distribution for each sample tested. In each plot, the curve labeled “Whole *E. coli*” corresponds to the side scatter distribution of whole *E. coli* cells, while the curve labeled “Particulates” corresponds to debris and other particulates that are not whole *E. coli* cells. (**a**) Side scatter distribution for the original sample which did not undergo treatment with the UV-C lamp; (**b**) Side Scatter distribution for a sample which underwent 15 min treatment with the UV-C lamp; (**c**) Side scatter distribution for a sample which underwent 1 min treatment with the UV-C lamp; (**d**) Side scatter distribution for a sample which underwent 5 min treatment with the ER.

Figure 5 illustrates the side scatter distribution for UV-treated *E. coli*, and shows that a degree of fragmentation occurs as a result of UV-C exposure. Figure 5b shows after 15 s of UV-C treatment, there is a small decrease (3489) in whole *E. coli* SSC counts, indicating UV-C cell damage causes slight fragmentation. Figure 5c,d shows that the number of whole *E. coli* SSC counts (~31,000), integrated under the curve, remain consistent between 5 min and 15 min exposure times. This data correlates with plate count and electrochemical detection, which indicate the partial sterilization of *E. coli* after 5 min of UV-C dosage. As expected, the mode of cell death deviates from ER treatment in that UV-C radiation leaves the cell relatively intact. 

### 3.2. Indirect Electrochemical Quantification of Inactivated E. coli O157:H7

Electrochemical SWV was used to quantitatively measure the amount of captured *E. coli* by indirectly measuring the amount of hybridized polyG tags. Damage to the membrane and DNA from ER and UV treatment has resulted in loss of attachment between the antibodies and *E. coli*, as indicated by electrochemical SWV. An initial scan (S1) measured the irreversible oxidation of polyG [12]. A second scan (S2) measured the baseline oxidation after the analyte was depleted, and was subtracted from S1 to find SWV curves.

**Figure 6:**
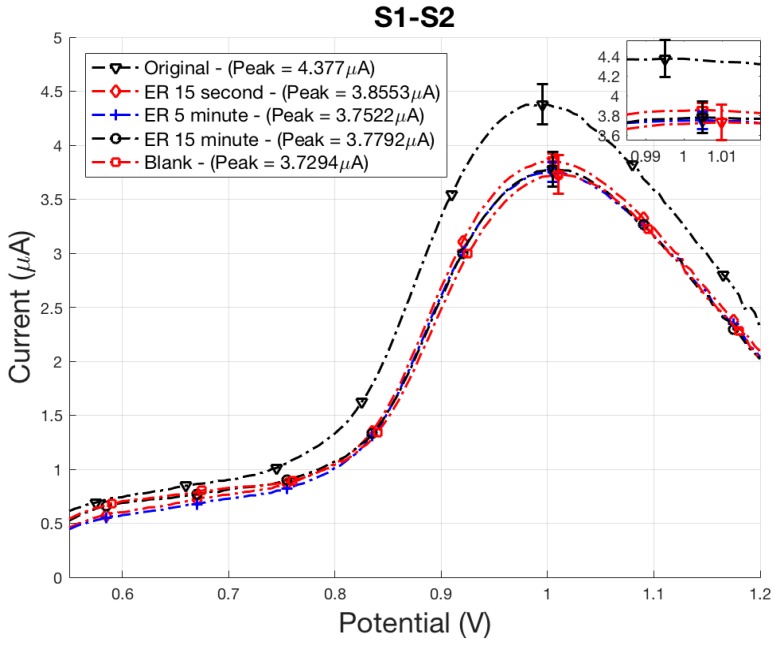
ER-treated *E. coli* electrochemical SWV curves displaying the oxidation of polyG tag.

Sensor results accurately identified the complete sterilization of *E. coli* cells using ER treatment as displayed in Figure 6. ER bactericidal effects were almost immediate, verified by the plated 15 min sample counting 0 cfu/mL. Quantification of *E. coli* is determined by comparing peak signal values to control peak signals, blank or original. If peak signal values for a sample are above the blank control, the sample contains *E. coli*. If peak values for a sample are the same as blank values, then the sample does not contain *E. coli*. As expected, peak signals for the 15 s, 5 min, and 15 min did not contain any viable cells. Peak signals for these samples were within reasonable experimental error of the blank control sample (Figure 6). The electrochemical sensor did not detect any viable cells in these samples because the bacteria in these samples were inactivated by ER and UV treatment. The sensor detects electrochemical polyG tags on the secondary bead, which are attached to the *E. coli* through receptors on the bacteria. If the receptors are unable to bind with the antibodies conjugated on the secondary bead, the electrochemical tag is not present during electrochemical sensing. After four IMS washes, damaged or fragmented membranes detach from the cell through mechanical mixing, breaking apart the assay necessary for detection. It is also likely that damage caused to the cell prevents receptors from attaching to antibodies on the magnetic and secondary bead.

**Figure 7:**
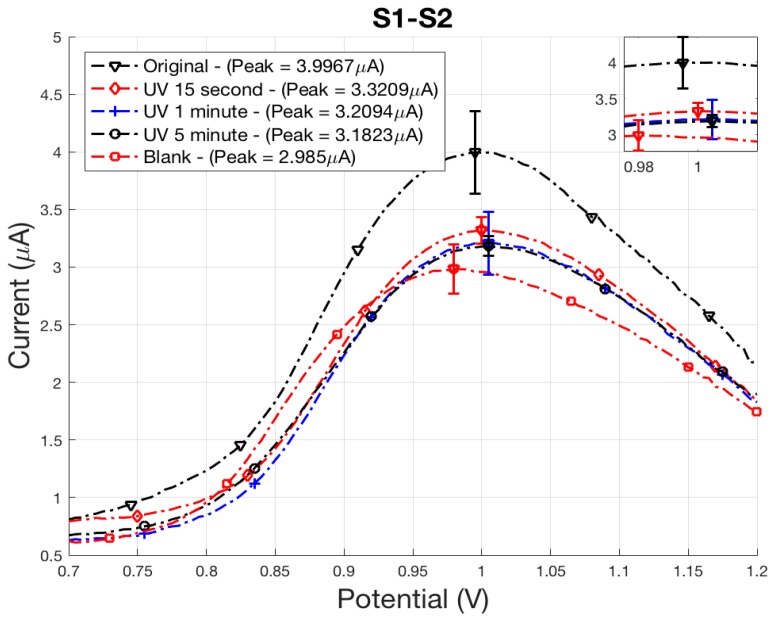
UV-treated *E. coli* electrochemical SWV curves displaying the oxidation of polyG tag.

Similar results occurred for *E. coli* inactivated using UV-C radiation, with peak signals falling between the positive and negative control samples, original and blank (Figure 7). An exposure time of 15 s resulted in a 0.67 µA oxidation peak reduction. Plate counts determined an average count of 20 cfu/mL were still viable, with a 2-log reduction of cells for a 15 min exposure time. With the exception of the 15 min treatment, signal strength for all ER treatment times fall within experimental uncertainty of the blank control sample. After 1 min of treatment, all plate counts were 0 cfu/mL indicating complete sterilization.

The sensor was successful in differentiating between active and inactive *E. coli*, even when the bacteria maintained its cell structure. As reported by Liu et al., UV radiation causes damage to the outer membrane where receptors would attach to the antibody [21]. This demonstrates that the cell membrane can remain intact, but damage to the receptors will prevent the senor from detecting sterilized bacteria. It is clear that damage done to the receptors prevents the formation of a magnetic bead/*E. coli*/polystyrene bead tag assay, and confirms the ability of the sensor to differentiate between active and sterilized cells.

## 4. Conclusions

The electrochemical sensor was able to correctly differentiate between viable and deactivated *E. coli* O157:H7 cells sterilized using both an electrocatalytic reactor and UV-C radiation. Plate counts and flow cytometry confirmed the reduction of viable cells and mode of bacteria death during ER and UV treatments. It is apparent that damage to receptor locations, through ER oxidation or UV-C exposure, prevented the attachment of *E. coli* to the antibodies and prevented polyG from reaching the electrochemical sensor after IMS washes. The magnetic bead/*E. coli*/polystyrene bead assays were not formed for *E. coli* cells physically destroyed in the ER, preventing the sensor from detecting an electrochemical signal from the polyG tag. 

## Figures and Tables

**Table 1:** TiO_2_-based electrocatalytic reactor-treated *E. coli*. O157:H7 electrochemical current density signal and plate counts.

Sample	Averaged Peak Signal (µA) (*n* = 3, ±SEM) *	Average Plate Counts (cfu/mL) (*n* = 3)
Original/No Treatment	4.37 (±0.18)	2.03 × 107 (±7.62 × 105)
15 s	3.86 (±0.07)	0
5 min	3.75 (±0.09)	0
15 min	3.78 (±0.16)	0
Blank Sample	3.73 (±0.18)	0

* *n* = sample size; SEM = standard error of the mean.

**Table 2:** Ultraviolet-C (UV-C)-treated *E. coli*. O157:H7 electrochemical signal and plate counts.

Sample	Averaged Peak Signal (µA) (*n* = 3, ±SEM) *	Average Plate Counts (cfu/mL) (*n* = 3)
Original/No Treatment	4.02 (±0.36)	50 × 106 **
15 s	3.32 (±0.11)	22 (±7.36)
5 min	3.21 (±0.27)	0
15 min	3.18 (±0.08)	0
Blank Sample	2.99 (±0.21)	0

* *n* = sample size; SE = standard error of the mean; ** OD600 measurements were used to verify counts.
